# Ultrasonic Coating of Poly(D,L-lactic acid)/Poly(lactic-co-glycolic acid) Electrospun Fibers with ZnO Nanoparticles to Increase Angiogenesis in the CAM Assay

**DOI:** 10.3390/biomedicines12061155

**Published:** 2024-05-23

**Authors:** Selina Streich, Julia Higuchi, Agnieszka Opalińska, Jacek Wojnarowicz, Pietro Giovanoli, Witold Łojkowski, Johanna Buschmann

**Affiliations:** 1 Medical Faculty, University of Zurich, Campus Irchel, 8006 Zurich, Switzerland; selina.streich@uzh.ch; 2 Plastic Surgery and Hand Surgery, University Hospital Zurich, 8091 Zurich, Switzerland; pietro.giovanoli@usz.ch; 3 Laboratory of Nanostructures, Institute of High Pressure Physics, Polish Academy of Sciences, Sokolowska 29/37, 01-142 Warsaw, Poland; j.higuchi@labnano.pl (J.H.); a.opalinska@labnano.pl (A.O.); j.wojnarowicz@labnano.pl (J.W.); w.lojkowski@labnano.pl (W.Ł.)

**Keywords:** ultrasonication, electrospinning, zinc oxide nanoparticles, angiogenesis, radical oxygen species, CAM assay

## Abstract

Critical-size bone defects necessitate bone void fillers that should be integrated well and be easily vascularized. One viable option is to use a biocompatible synthetic polymer and sonocoat it with zinc oxide (ZnO) nanoparticles (NPs). However, the ideal NP concentration and size must be assessed because a high dose of ZnO NPs may be toxic. Electrospun PDLLA/PLGA scaffolds were produced with different concentrations (0.5 or 1.0 s of sonocoating) and sizes of ZnO NPs (25 nm and 70 nm). They were characterized by SEM, EDX, ICP-OES, and the water contact angle. Vascularization and integration into the surrounding tissue were assessed with the CAM assay in the living chicken embryo. SEM, EDX, and ICP-OES confirmed the presence of ZnO NPs on polymer fibers. Sonocoated ZnO NPs lowered the WCA compared with the control. Smaller NPs were more pro-angiogenic exhibiting a higher vessel density than the larger NPs. At a lower concentration, less but larger vessels were visible in an environment with a lower cell density. Hence, the favored combination of smaller ZnO NPs at a lower concentration sonocoated on PDLLA/PLGA electrospun meshes leads to an advanced state of tissue integration and vascularization, providing a valuable synthetic bone graft to be used in clinics in the future.

## 1. Introduction

With a surge in performed procedures and contemporaneous scientific progress, there is a growing demand for artificial scaffolds in reconstructive medicine [1,2,3]. Scaffolds made from resorbable biopolymers enable the endogenous regeneration of tissue defects [4]. Mimicking the target tissue’s extra cellular matrix, they temporarily provide biomechanical stability, and are then decomposed and resorbed as the newly formed tissue is being remodeled [5]. Their properties, including stability, size, and shape, can be designed according to their area of application [6]. Hence, they present a valuable long-term alternative to auto- and allografts, with advantages regarding supply, site-specific design, no donor site morbidity, pathogen transmission, and immunorejection, and, compared to traditional, permanent implants, there is no concern for erosion [7].

To attain successful tissue integration within the scaffold, its architecture and surface need to promote cell adhesion, proliferation, and migration, allow the permeation of nutrients and metabolites, and be conducive to vascularization [8]. Porous scaffolds produced through electrospinning from poly(D,L-lactic acid) (PDLLA) and poly(lactic-co-glycolic acid) (PLGA) solutions show good biocompatibility, little to no foreign body reaction, and an adjustable degradation time [9,10,11,12]. The electrospun fiber network is favorable for cell attachment and growth and provides a mechanical template for guided tissue repair [13]. Moreover, thin electrospun membranes are to be used to substitute avascular tissues like skin or cartilage [13]. However, to sustain tissue growth beyond the oxygen diffusion limit of 100–200 μm, the formation of a capillary network throughout the scaffold is of paramount importance [14]. The spontaneous vascularization of a scaffold after implantation in vivo is limited to tenths of micrometers per day [15], resulting in insufficient transport of oxygen, nutrients, metabolites, and cells involved in tissue remodeling to the interior of the scaffold, eventually causing implant failure [16].

Here, nanotechnology can be used to modify the scaffold surface promoting angiogenesis. Different approaches have been investigated, with varying complexity degrees in manufacturing and vastly different shelf-lives of the scaffolds [17,18]. Recently, studies have investigated the angiogenic potential of zinc oxide (ZnO) nanoparticles (NPs) in different forms and applications, with both pro-angiogenic [19,20,21,22,23] and anti-angiogenic [24,25] reports. Augustine et al. suspected the angiogenic effect to be dependent on the ZnO NP concentration [20]. ZnO NPs induce reactive oxygen species (ROS), which activate vascular endothelial growth factor (VEGF) expression and stimulate cell proliferation, migration, and tube formation for angiogenesis [20,26]. ZnO NPs are already widely used in tissue engineering due to excellent biocompatibility and degradability, promotion of cell attachment and proliferation, osteoconduction, and their antibacterial properties, hence improving both tissue integration and the issue of implant infection [27]. In contrast, there are studies that have associated ZnO NPs with cytotoxicity and inflammation [28].

ZnO NPs can be obtained by different chemical methods, such as hydrothermal synthesis [29], sonochemistry [30], solvothermal synthesis [31], the sol–gel method [32], and mechano-chemical synthesis, exemplified by ZnO nanowires [33]. On the other hand, ZnO NPs can be produced by green synthesis [34], for example, from *Ilex paraguariensis* [35] or *Prosopis cineraria* leaf extracts [36]. Combined with different polymers, ZnO NPs have been successfully applied in wound healing, realized as a PLGA/silk fibroin/ZnO NP antibacterial wound dressing [37], and as alginate hydrogels with embedded ZnO NPs leading to higher stiffness, increased humidity retention, and bactericidal activity, but not affecting cell viability [38]. Moreover, chitosan/pectin/ZnO NP scaffolds showed that fibroblasts grew very well in addition to promoting antimicrobial activity against Gram-positive and -negative bacteria [39]. Further biopolymers with incorporated ZnO NPs have been reported, like chitosan/poly vinyl alcohol/nZnO [40], chitosan/poly caprolactone/nZnO [41], and PLGA/VEGF/nZnO [23], respectively. Finally, a recent comprehensive review has summarized biocomposites with ZnO NPs [42]. 

Originally developed by Prof. Domenico Ribatti [43,44,45], the chorioallantoic membrane assay (CAM assay) of the chicken embryo is a cost-effective model to study angiogenesis in ovo. The CAM assay is not only designed for studying angiogenesis but also tumor biology [46], vascularization of acellular biomaterials [47] and organoids [48], and high-throughput preclinical imaging [49,50,51].

The main goal of this study was to conclusively show the pro-angiogenic potential of microdosed ZnO NPs in a biopolymer scaffold as a function of NP size and concentration. To precisely control the size of the nanoparticles and ensure their high crystallinity degree the recently developed Microwave Solvothermal Synthesis technology (MSS) that permits the synthesis of nanoparticles with a size regulated from 20 to 120 nm was exploited [52,53]. For this purpose, ZnO NPs were deposited on the surface of an electrospun PDLLA/PLGA scaffold, using the ultrasonic coating method [12,54]. As both the NP size and surface area exposed can influence the concentration present, two different ZnO NP sizes (A—25 nm, specific surface area (SSA) = 48 m^2^/g and B—70 nm, SSA = 16 m^2^/g) and two different sonocoating times (0.5 and 1.0 s) were tested using the CAM assay, resulting in four different NP concentrations to be compared to a plain scaffold (no ZnO NPs; control). 

## 2. Materials and Methods

### 2.1. Chemicals

PDLLA (PURASORB^®^ PDL 20) and PLGA (PURASORB^®^ PDLG 5010) polymers were supplied for fabrication of electrospun membranes in GMP grade by Corbion, Amsterdam, Netherlands. 2,2,2-Trifluoroethanol (TFE) solvent for the electrospinning of fibers was purchased from Sigma-Aldrich, St. Louis, MO, USA. ZnO NPs (25 and 70 nm) were synthetized by using microwave solvothermal method [52,53] and supplied by Laboratory of Nanostructures, IHPP PAS.

### 2.2. Electrospinning

**Figure 1:**
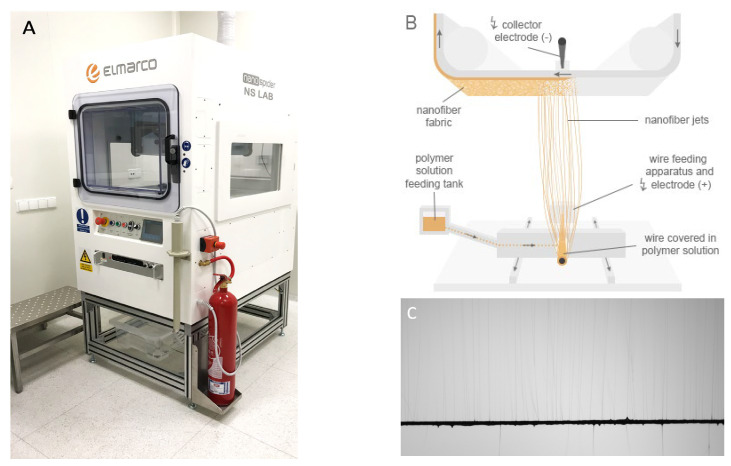
Electrospinning process using the Elmarco Nanospider NSLAB device (**A**), illustrated in (**B**). Digital camera view on the magnified wire producing fibers (**C**).

Polymer granules of PDLLA (5 g) and PLGA (5 g) were dissolved in 50 mL of 2,2,2-TFE solvent. The resulting transparent solution of 20% concentration was obtained by stirring at magnetic stirrer for 24 h in ambient conditions. For the purpose of electrospinning the NSLAB device (Elmarco, Liberec, Czech Republic, Figure 1) was used in cleanroom class ISO 8 facilities to provide a dust-free environment. Process parameters were set to voltage of 30 kV, 25 cm distance to substrate, and spinning carriage speed of 1 s 35 cm. As a collecting substrate, spunbonded fabric was used.

### 2.3. Ultrasonic Coating of Membranes: Sonocoating

**Figure 2:**
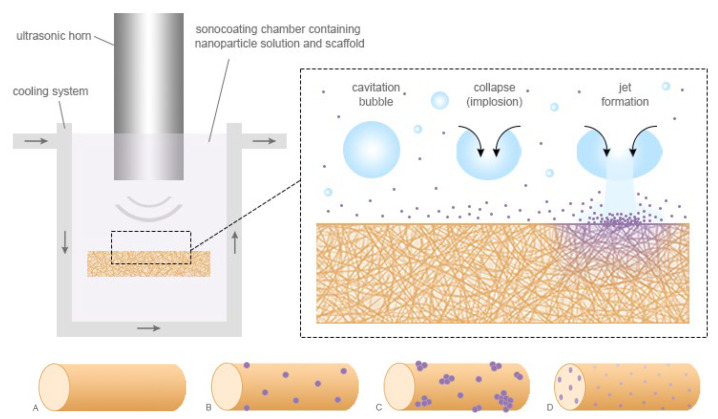
Scheme of the sonocoating process. The scaffold is placed in the solution containing nanoparticles. Ultrasonic waves lead to the formation of cavitation bubbles, which penetrate the fibrous structure and whose subsequent implosion deposits ZnO NPs on its surface. Illustration (**A**) shows a plain electrospun polymer fiber, (**B**,**C**) illustrate sonocoated fibers with two different deposition times of NPs and fiber, and (**D**) depicts a different approach, chosen, e.g., by Augustine et al., where NPs are incorporated into the polymer solution before electrospinning, with most of their surface located within the fiber as a result.

For the process of NP deposition on fibers, the patented ultrasonic coating method was used [55,56]. Samples were cut into 4 cm × 4 cm squares and fixed in either the solution of 25 nm or 70 nm ZnO NPs. Subsequently, ultrasonic cavitation wave was induced in the vicinity of the sample. The solution used for fabrication of samples was 0.1% of ZnO NPs in deionized water. Ultrasonic coating was performed for 0.5 and 1.0 s, deposition times preselected in a series of prior optimization trials (2, 4, 6, 8 and 10 s; Appendix A, Table A1), respectively, on lab-scale setup equipped with UP100H Hielscher ultrasonic homogenizer and water-cooling system (Figure 2). After the deposition, samples were rinsed before drying overnight in a laminar flow cabinet. In contrast to sonocoated fibers (Figure 2B,C), blends of ZnO NPs with a polymer lead to fibers with incorporated NPs homogenously distributed in the corresponding fibers (Figure 2D).

### 2.4. Characterization

Scanning electron microscopy (SEM). The morphology of the fibrous membranes with and without ZnO NPs was analyzed using SEM (Ultra Plus GEMINI, Carl Zeiss, Jena, Germany). Prior to imaging, the samples were sputtered with a layer of carbon with an average thickness of 10 nm to ensure the flow of charge from the sample surface to the stage. The measurements were taken at an accelerating voltage of 2 kV. Following imaging, measurements of each sample type were completed using ImageJ ver. 1.52 (NIH, Bethesda, MA, USA). For this method, the scale bar was set with respect to the image pixel size. The average size of material’s pores was measured by tracing the outer area of randomly chosen pores (*n* = 50).

Images of the surface topography of the samples were recorded using a VHX-7000 digital microscope equipped with a DIC varifocal lens (VH-Z100UT) for automatic measurement of the roughness profile (Keyence Europe, Mechelen, Belgium). Surface roughness was determined from cross-sectional linear profiles derived from three-dimensional images placed at the center of the top surface of the samples (*n* = 10). A 3D reconstruction of the surface topography was performed using depth composition for full imaging sharpness.

Energy-dispersive X-ray (EDX) analysis. The qualitative microanalysis of the surface of the samples was performed using a scanning electron microscope (Ultra Plus GEMINI, Carl Zeiss, Germany) equipped with the EDX Quantax 400 analysis system (Bruker, Billerica, MA, USA). Based on the analysis, the elemental composition of the surface was identified. The measurements were taken at an accelerating voltage of 30 kV.

Static water contact angle (WCA). WCA measurement was performed using a goniometer (model DSA25B, Krüss, Germany). The measurement consisted of observing a drop of deionized water (conductivity of 0.09 μs/cm) placed on the sample and directly measuring the angle at the contact with the surface. Drops with a volume of 10 μL were applied to the sample surface which was perpendicular to the dosing needle. The shape of the droplet was recorded by a digital camera and processed using the Krüss ADVANCE computer program (Krüss, Hamburg, Germany). The Young–Laplace equation was used in the analysis of the droplet shape. The WCA measurement was carried out on uncoated and sonocoated membranes, with various amounts of ZnO NP. 

Inductively coupled plasma optical emission spectroscopy (ICP-OES) analysis was performed on Optima 8300 device supplied by Perkin Elmer, Shelton, CT, USA. The exact amount of ZnO NPs deposited on the membranes was assessed by ICP-OES. Samples were prepared by microwave-assisted digestion in HNO_3_ acid for 20 min at 210 °C in Ethos Up, Milestone Srl, Sorisole, Italy, before analysis.

### 2.5. Chicken Chorioallantoic Membrane (CAM) Assay

**Figure A1:**
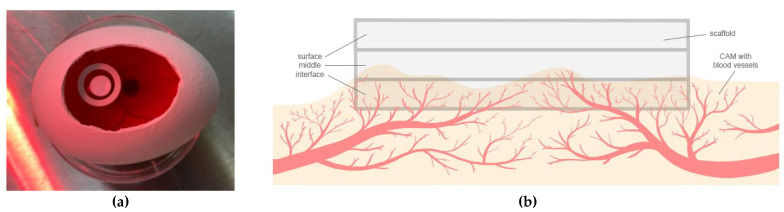
(**a**) Placement of the scaffold onto the CAM on EDD 8. The scaffold is positioned inside a placeholder ring. The developing eye and blood vessels are well visible underneath. (**b**) Schematic section of the scaffold illustrating the zone division.

To investigate the angiogenic potential of the scaffolds sonocoated with ZnO NPs, the CAM assay has been carried out in ovo. This method is standard for testing angiogenic activity and biocompatibility in vivo [47]. Fertilized chicken eggs were incubated horizontally at a temperature of 37 °C. On embryo development day (EDD) three, 5 mL of albumin was removed from the blunt end of the egg. This way, the developing CAM detached from the shell and the underlying CAM vessels were disclosed. Then, an elliptical patch of eggshell of approximately 2.5 × 3.5 cm^2^ was removed and the hole covered with a Petri dish to prevent evaporation. The egg was labeled and returned into the incubator in a static position. On EDD eight, the scaffolds were placed onto the CAM (Appendix A, Figure A1a), and then the eggs were again covered, labeled, and returned to the incubator in a static position. After one week (EDD 15), formalin was injected under and spread over the scaffolds on the CAM for fixation. The eggs were subsequently transferred to the refrigerator at 4 °C overnight. The next day, photographs were taken from above, the scaffolds were excised, placed upside down onto a Petri dish, and photographed again. 

### 2.6. Histological Analysis

For histological analysis, the fixated scaffolds were then cut in half, embedded in paraffin, and stained. Hematoxylin–Eosin and Masson–Goldner Trichrome staining was used. Analysis of the histological section was focused on the area of the scaffold. Vessel ingrowth could take place from the interface, the sides, or in case of overgrowth also from the surface, i.e., when the scaffold was completely absorbed by the CAM. Each sample was divided into three equally distanced zones: surface, middle, interface (to the CAM, Appendix A, Figure A1b). Five representative fields of view (FOVs) were chosen per zone and manually quantified according to predefined parameters. As parameters for vascularization, the number of vessels per area (number of vessels per 100 × 100 μm^2^) and the area of vessels per area (area of lumen (μm^2^) divided by area of FOV (100 × 100 μm^2^)) were determined. To detect further effects of the NP, the cell count (number of all cells per area of FOV (100 × 100 μm^2^)) and tissue integration (distance from CAM surface to front of tissue infiltration (μm)) were quantified.

### 2.7. Statistical Analysis

The software SPSS (IBM, Armonk, NY, USA, IBM Statistics Version 27.0) was used for descriptive statistics, analysis of variance (ANOVA) and to represent the results in boxplots. Post hoc Bonferroni tests allowed a comparison for significance between the groups. A *p* value less than 0.05 was considered as statistically significant. Statistical significances are marked with * (*p* < 0.05), ** (*p* < 0.01) and *** (*p* < 0.001). 

## 3. Results

### 3.1. Membranes Characteristics and Morphology

**Figure 3:**
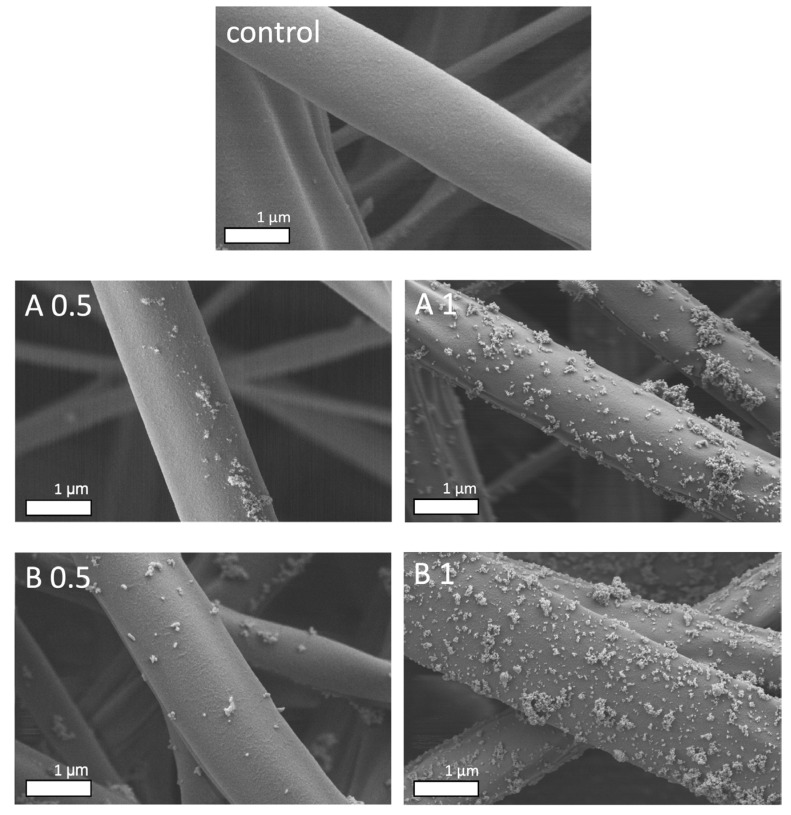
SEM images showing the uncoated PDLLA/PLGA fibers (**top**, control) and the fibers sonocoated with ZnO NP group A = 25 nm (**middle row**) and group B = 70 nm (**bottom row**). Key: A 0.5 = group A sonocoating time 0.5 s.

SEM imaging confirmed that the deposition of ZnO NPs of both sizes was successful. Figure 3 shows fibers covered with scattered groups of NP, resulting from the shortest sonocoating process conducted for 0.5 s, and more homogeneously covered fibers with deposition times of 1 s. Hence, four groups combined from two different NP sizes (A = 25 nm, B = 70 nm) and two different sonocoating times (0.5 or 1 s) could be produced with their total amounts of ZnO NPs varying from 0.1 to 0.24 wt %. (Table 1). 

**Figure 4:**
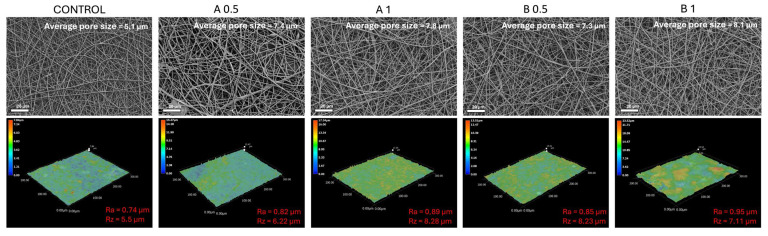
SEM images of the samples and their corresponding surface topography profiles.

The result of ImageJ pore size analysis of the samples revealed that all the samples showed comparable structure. The process of sonocoating did not cause the destruction of the material. Nevertheless, the average pore sizes and surface roughness values of sonocoated samples were slightly bigger in comparison with the control samples (Figure 4) due to the penetration of the structure with NPs during the sonocoating process in water suspension of ZnO NPs.

### 3.2. Water Contact Angle 

**Figure 5:**
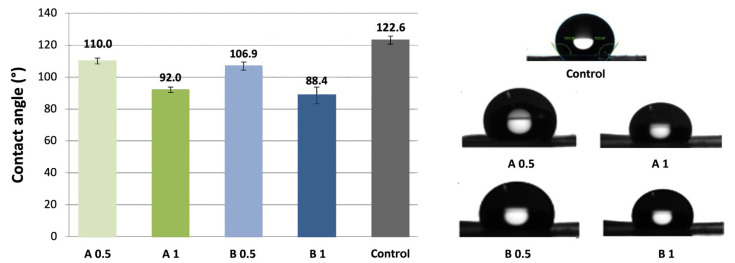
Water contact angles for the samples of groups A and B with two different sonocoating times (0.5 and 1.0 s) and control. Above each bar, the WCA value is presented in °; for example, it is 110.0 ° for group A 0.5.

The obtained water contact angles for the samples (A 0.5, A 1, B 0.5, B 1, and control) were measured and results indicated changes in surface wettability (Figure 5). It was noticed that even minor surface modification of 0.5 s ZnO NP deposition resulted in the lowering of contact angles in comparison with the control PDLLA/PLGA sample.

### 3.3. Chemical Composition

**Figure 6:**
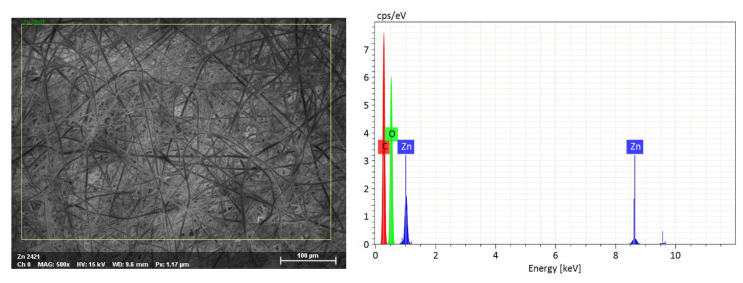
SEM image and EDX spectra of PDLLA/PLGA fibers sonocoated with ZnO NPs (group A 1).

Figure 6 represents the EDX-mapping spectra of synthesized membranes of A 1 group. The respective analysis revealed the occurrence of two peaks. The obtained peaks correspond very well with the major emission energies of zinc and oxygen. Spectra confirmed the successful inclusion of ZnO NPs on the surface of PDLLA/PLGA fibers. No differences between A and B groups were detected.

### 3.4. Impact of ZnO NPs on Angiogenesis in Ovo

**Figure 7:**
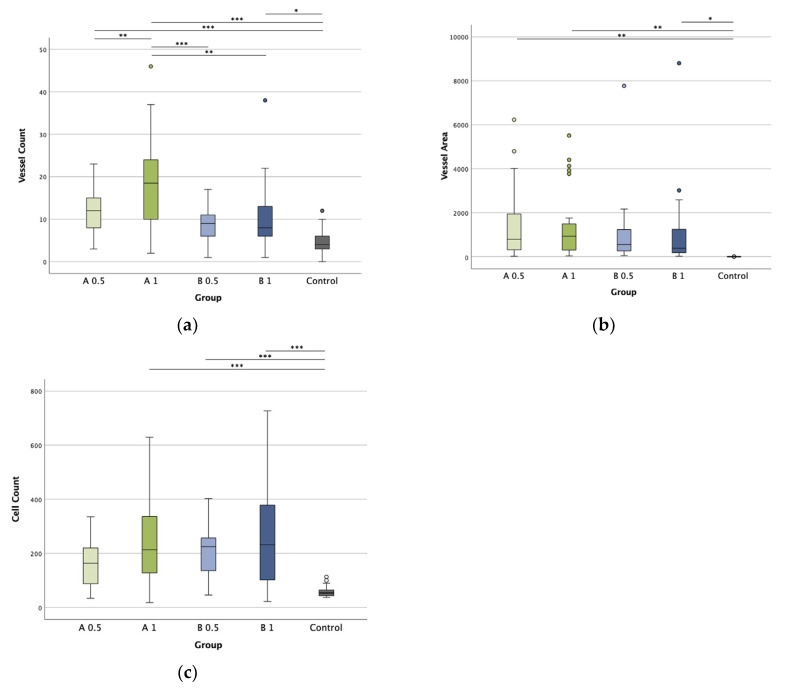
Vascularization and cell infiltration. Vascularization, assessed through vessel count/(100 μm)^2^ (**a**), vessel area/(100 μm)^2^ (**b**), and cell count/(100 μm)^2^ (**c**). Control represents the plain polymer scaffold electrospun from 50% PDLLA and 50% PLGA without ZnO NPs. The names of the four sonocoated groups describe the ZnO NP size (A = 25 nm, B = 70 nm) and the duration of ultrasonic coating (0.5 or 1 s), respectively. Key: *p*-values ≤ 0.05 were considered significant and denoted with (*), for *p*-values ≤ 0.01 (**), and *p*-values ≤ 0.001 (***).

To assess angiogenic properties of ZnO NPs, we compared the four groups containing ZnO NPs to the plain biopolymer scaffold with respect to vessel count and vessel area. Higher sonocoating times leading to higher ZnO NP concentrations ( Appendix, Table A1) were not tolerated in the sensitive CAM assay. Figure 7 shows the overall vessel density and vessel area, clearly underlining the ZnO NPs’ pro-angiogenic properties in both aspects. 

**Figure A2:**
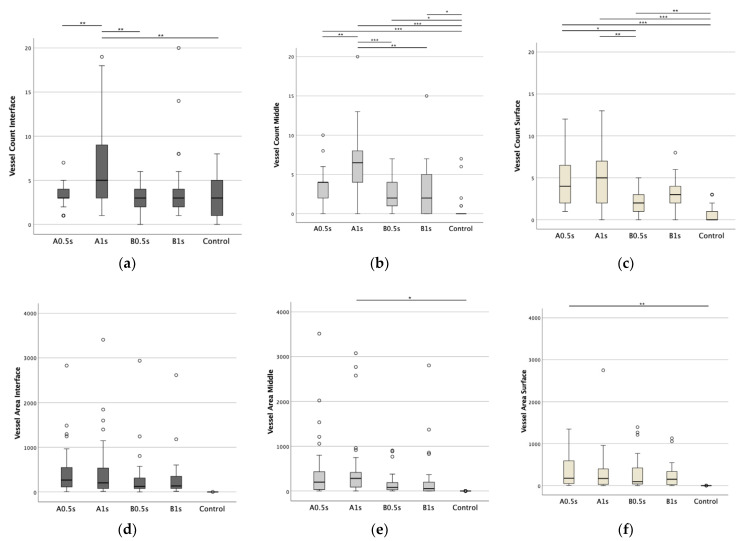
Vessel count (**a**–**c**) and vessel area (**d**–**f**). The division of the scaffold sections into three layers (interface, middle, surface) facilitates the assessment of the impact of ZnO NPs and their respective differences in detail. Key: *p*-values ≤ 0.05 were considered significant and denoted with (*), for *p*-values ≤ 0.01 (**), and *p*-values ≤ 0.001 (***).

To assess vascularization throughout the scaffolds, we further divided the scaffold’s histological sections into three layers (interface, middle, and surface). This way, we could specifically show the significantly increased vascularization of the middle and surface layer in ZnO NP-containing scaffolds (Appendix A, Figure A2). The plain biopolymer scaffold showed a rapid decrease in vascularization with increasing distance from the contact area with the CAM. The vessel density in the interface layer was comparable to the ZnO groups but very little to no vessels could be found in the middle and surface area. 

While all scaffolds sonocoated with ZnO NPs showed an overall higher vessel density and area compared with the control, group A 1 showed a significantly higher vessel count compared to the other ZnO NP-containing groups and was the only group with a significantly increased vessel density in the interface layer. Group A 0.5 showed a lower vessel count. Vessel areas/area were similar among the ZnO NP-containing scaffolds and only significantly higher compared to the control; consequently, group A 0.5 with a lower vessel density exhibited larger vessels. In comparison with the control, the two A groups showed the greatest significances, while group B 0.5 showed the least or no significant differences in vascularization. 

### 3.5. Cell Count in Presence of ZnO NPs

We assessed the cell count identically to the vessel count. As shown in Figure 7c, all groups sonocoated with ZnO NPs showed significantly higher cell count, with the highest values in the 1 sec groups throughout all layers. As a trend, shorter sonocoating times (lower ZnO NP concentrations) exhibited lower cell densities. 

### 3.6. Tissue Integration

**Figure 8:**
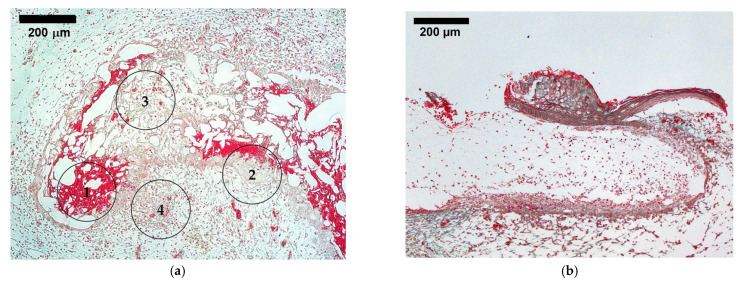

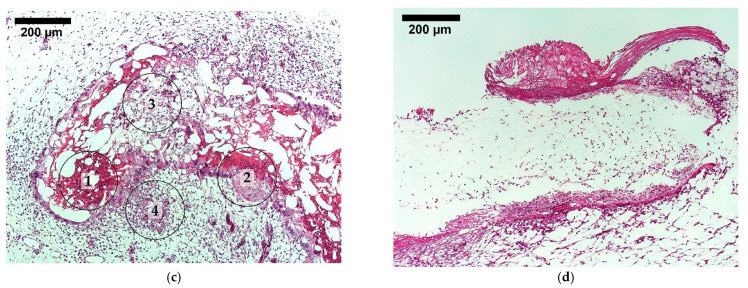
Masson–Goldner Trichrome-stained histological section of a scaffold fully tissue integrated from the A 1 group (**a**). Here, different stages of the regeneration process can be recognized. Area **1** shows an eosinophile, hypercellular scaffold area; in **2**, granular tissue with macrophages, resorption vacuoles, and fibrin deposits can be seen; while in **3**, there is already a partly remodeled soft tissue visible, showcasing collagen and many vessels. Circle **4** marks a hypercellular and -vascular part of the CAM surrounding the scaffold. In contrast, the control showed only a scarce amount of cells present further than 100 μm from the CAM intersection (**b**). For comparison, Hematoxylin–Eosin-stained serial section, with (**c**) showing the same excerpt as in (**a**) and with (**d**) reflecting the same area as (**b**). Scale bars indicate 200 μm.

When histological sections were analyzed as exemplified for one typical Masson–Goldner Trichrome stained section of the A 1 group (Figure 8a), the ZnO NP-containing scaffolds were often overgrown with CAM tissue and reflected several stages of tissue regeneration in one and the same sample: Hypercellular areas as encountered during initial tissue ingrowth (region 1), granular tissue (region 2), remodeling for an advanced state (region 3), and finally the hypercellular and hypervascular part of the adjacent CAM (region 4). In contrast, when scaffolds without NPs were inspected, in most of the cases, no tissue overgrowth had taken place and most of the core scaffold was not infiltrated by tissue (Figure 8b,d). The position of the scaffold in Figure 8b,d shows that the CAM is below the scaffold, but that on the right side of the image, some tissue has started to encapsulate the scaffold. 

## 4. Discussion

Electrospun biopolymer scaffolds appear to be a valuable long-term solution for the endogenous repair of tissue defects and their use as membranes for avascular tissue replacements is being extensively explored [5,6,13]. Vascularization of such scaffolds, enabling tissue integration beyond the oxygen diffusion limit, is now a crucial challenge to solve to promote their use as soft tissue substitutes in larger, clinically relevant sizes [57]. Here, nanotechnology offers the option of stimulating a desired tissue reaction by means of additive biomanufacturing. ZnO NPs have been broadly investigated for tissue-engineering purposes and have proven to be valuable additives promoting cell adhesion, proliferation, and differentiation, preventing implant infection with antibacterial properties while exhibiting excellent biocompatibility and degradability [27]. However, even in recent studies, their pro-angiogenic properties within scaffolds could not conclusively be established [58]. 

In the scope of this study, we fabricated and characterized electrospun PDLLA/PLGA scaffolds with sonocoated ZnO NPs. SEM images confirmed the coating of NPs on the electrospun fibers. EDX and ICP-OES revealed the presence of ZnO in these scaffolds. The higher the ZnO NP concentration was, the more hydrophilic the surface of the meshes, as assessed by water contact angle, independently of the NP size. We furthermore demonstrated a significantly improved vascularization in presence of ZnO NPs. Using the CAM assay, a typical model system to study angiogenesis [48,50,51,56,59], we compared four groups with different concentrations of ZnO NPs, constructed from two different NP sizes (A = 25 nm, B = 70 nm) and two different sonocoating times (0.5 or 1 s), to the plain PDLLA/PLGA biopolymer scaffold. Previous studies have shown the angiogenic properties of ZnO NPs by analyzing the CAM and CAM-scaffold interface vessels [20,58]. In this study, however, we were able to show the sprouting of vessels throughout the different scaffold layers via the histological analysis of scaffold sections. 

The scaffolds containing ZnO NPs showed increased vascularization, particularly expressed in the upper two thirds of the scaffold, where the plain biopolymer scaffold showed little to no vessels, similarly to findings reported for chitosan/cellulose hydrogels with ZnO NPs applied in the CAM assay [60]. This correlates with the finding that the spontaneous sprouting of vessels into a scaffold is very slow, limited to tenths of micrometers per day [15]. ZnO NPs are known to generate ROS, specifically superoxide ions [22]. They are important players in redox signaling pathways for angiogenic responses in physiological repair processes [61], e.g., ischemia-induced angiogenesis [62], as wells as in pathological vessel growth, e.g., in tumors and dermatoses [63]. Neovascularization occurs via angiogenesis, the sprouting of capillaries from existing blood vessels, and vasculogenesis, the de novo assembly of vessels from endothelial progenitor cells [18]. They are induced by different signaling pathways, which are themselves orchestrated through a complex interplay of growth factors, cytokines, and microenvironmental cues [17]. The dominant actor amongst them is the VEGF [64]. In VEGF-dependent angiogenesis, ROS promote VEGF expression and secretion by the up-regulation of hypoxia-induced factor 1α (HIF-1α), potentiate the VEGF receptor 2 (VEGFR-2) phosphorylation, and are required in the subsequent downstream signaling of PI3K/AKT and MAPK pathways [65,66,67]. This eventually leads endothelial progenitor cells to migrate towards the factor gradient, and promotes cell assembly, vessel formation, and maturation [68]. The histological sections of the scaffolds impressively depict the impact of successful vascularization on tissue integration and, hence, the success of ZnO NPs in a scaffold. The pure polymer scaffolds in the control group exhibited a band-shaped tissue integration (Figure 8a) with a mean tissue integration of 77 ± 11 μm and a maximum distance of 255 μm away from the CAM. This correlates with the oxygen diffusion limit of 100–200 μm [14]. In contrast, the scaffolds sonocoated with ZnO NPs were fully integrated and even showed all stages of wound healing, including remodeling (Figure 8b).

Apart from stimulating angiogenesis, ROS also have antibacterial and cytotoxic properties, making them a valuable additive for implant infection prevention [58] and tumor treatment [69]. The critical parameter determining whether ZnO NPs exhibit these adverse or the before-mentioned regenerative effects on tissues seems to be the concentration of induced ROS. Higher amounts of ROS are useful or even needed for cytotoxic and bacteriostatic effects, as shown in a study where ZnO NPs were directly put on the CAM surface (no biocomposite) [70] or with gelatin-coated ZnO NPs [71], while the dose to achieve a pro-angiogenic response is much lower and its range narrower [65]. In consideration of this, it is important to understand how the generation of ROS is influenced by ZnO NPs to use them accordingly. ROS are produced as cells are exposed to ZnO NPs, mainly by the nicotinamide adenine dinucleotide phosphate (NADPH)-oxidase enzyme system following incomplete phagocytosis [66]. Additionally, ROS arise when free radicals bind to the NP surface, or when ZnO NPs are dissolved, leading to free zinc ions [66]. The latter has been described as the lysosome-enhanced Trojan horse effect [72], with intracellular cytotoxic effects occurring as zinc ions are released during the lysosomal dissolution of ZnO NPs. Hence, apart from the concentration, both the size and shape of ZnO NPs have a significant impact on the amount of ROS generated. ZnO NPs with a high specific surface-to-volume ratio, which is the case for small NPs, show more prominent ROS generation [73]. In studies investigating biological impact and cytotoxic effects, the optimal size range for ZnO NPs was found to be around 30–50 nm, regardless of the NP composition, as these sizes had the highest uptake rate and intracellular accumulation via passive diffusion [74]. Additionally, the surface charge of ZnO NPs has been identified as a critical parameter affecting ROS generation, with positively charged NPs generating the highest concentrations of ROS [75]. 

To validate these findings for angiogenic purposes, we compared smaller and larger ZnO NPs in their unmodified state, hence featuring a positive surface charge in aqueous solutions, applied in microdoses (0.1 up to 0.24% wt). The groups containing the smaller-sized NPs exhibited the highest values in vascularization parameters, with the scaffolds sonocoated for one sec (0.24% wt) with these smaller 25 nm ZnO NPs showing a significantly higher vessel density throughout all scaffold layers compared to all other groups. Interestingly the group sonocoated for only 0.5 s (0.10% wt) displayed a lower vessel density but a similar or even higher vessel area, which already indicates a progression to vessel maturation with larger diameters. This result is consistent with the previous findings and allows us to conclude that a smaller-sized 25 nm ZnO NPs, sonocoated in a microdose concentration onto the scaffold, induces an optimal, namely sufficient yet not toxic, amount of ROS for the desired pro-angiogenetic effect. The comparison of the four groups also impressively illustrates the high sensitivity of the tissue, or in turn, the impact of NP size and an already 0.6% wt difference in the ZnO NP concentration.

As for the cell count, all sonocoated groups had higher values compared to the plain scaffold, with the two groups sonocoated for one sec attaining the best values and no significant differences between the two NP sizes. This enhancement is also driven through the presence of ZnO NP-induced ROS. In moderate amounts, they stimulate, amongst others, fibroblast growth factor 2 (FGF-2) and thereby enhance cell attachment, proliferation, and migration [76,77]. Another important criterion for cell adhesion is the scaffold’s surface. Sonocoating is a technique where the scaffold is suspended in an NP solution, and via ultrasonic input the dispersed NPs get thrown onto the fiber’s surface [78], adhering in carpet-like clusters [79]. With longer sonocoating times, more NPs get deposited on the surface, creating more comprehensive areas of NPs, subsequently turning the perfectly smooth surface of electrospun fibers into a rough nanotopography. This supports an early cell–biomaterial interaction and tissue integration through improved hydrophilicity and water absorption and supports cell proliferation [54]. Compared to scaffolds incorporating 1% wt. ZnO NPs into the biopolymer solution before electrospinning [20], this technique achieves a boosting effect for tissue integration with much smaller doses of ZnO NPs. As they are exposed on the fiber surface only, they amplify regenerative signaling in the initial phase of wound healing without causing additional oxidative stress during the scaffold’s resorption. Sonocoating is also a favorable technique regarding the manufacturing process due to short coating times, low coating temperature, water as a solvent, low NP concentrations needed for the solution, and tunable parameters, allowing an easy upscale [80].

## 5. Conclusions

We were able to demonstrate that a sonocoated surface with ZnO NPs promotes vascularization and integration compared to pure PDLLA/PLGA electrospun meshes, except for the group with 70 nm NPs (group B) and shorter sonocoating time. We conclude that smaller-sized 25 nm ZnO NPs (group A) are more pro-angiogenic compared to larger NPs with a size of 70 nm because the smaller NPs evoked fewer, but larger vessels, which indicates a more developed and progressed state of angiogenesis. It was shown that sonocoating is an efficient technology to deposit the ZnO NPs on the fibers and modify their properties. As for the concentration, a sonocoating time of 0.5 s revealed a more advanced level of tissue response, with lower cell numbers, suggesting that the hypercellular state usually observed during tissue integration of implants had been already over. Our study paves the way for such bone void fillers to be tested further in small animal models and then be translated into the clinical setting in the future. 

## Figures and Tables

**Table 1:** Overview of fabricated samples.

Sample Group Name	Size of ZnO NP	Deposition Time	Amount of ZnO NPs *
A 0.5	25 nm	0.5 s	0.10 wt%
A 1	25 nm	1 s	0.24 wt%
B 0.5	70 nm	0.5 s	0.12 wt%
B 1	70 nm	1 s	0.18 wt%

* Amount of zinc oxide measured by ICP-OES method.

**Figure A3:**
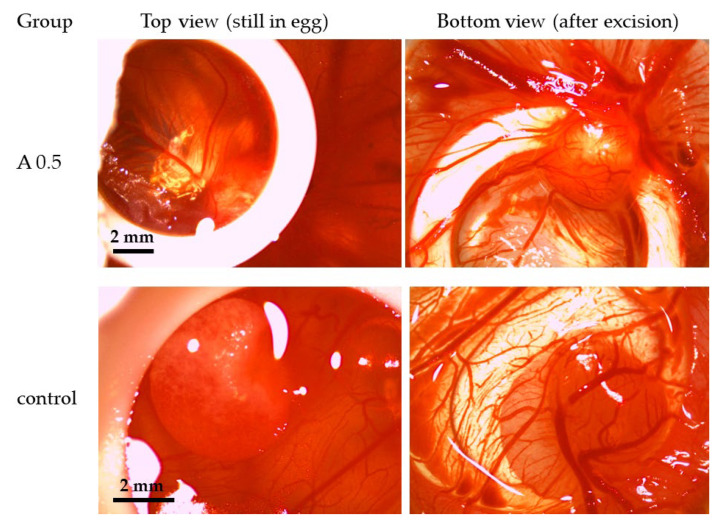
Typical examples of vascularized scaffolds in the CAM assay before extraction viewed from top (**left**) and after excision photographed from bottom view (**right**).

## Data Availability

The raw data supporting the conclusions of this article will be made available by the authors on request.

## Appendix A

**Table A1:** All fabricated samples. * Amount of zinc oxide measured by ICP-OES method.

Sample Name	Type of Nanoparticles	Deposition Time	Amount of ZnO *
A 0.5	ZnO 25 nm	0.5 s	0.1 wt%
A 1	ZnO 25 nm	1 s	0.24 wt%
A 2	ZnO 25 nm	2 s	0.50 wt%
A 4	ZnO 25 nm	4 s	0.61 wt%
A 6	ZnO 25 nm	6 s	0.73 wt%
A 8	ZnO 25 nm	8 s	0.80 wt%
A 10	ZnO 25 nm	10 s	1.01 wt%
B 0.5	ZnO 70 nm	0.5 s	0.12 wt%
B 1	ZnO 70 nm	1 s	0.18 wt%
B 2	ZnO 70 nm	2 s	0.45 wt%
B 4	ZnO 70 nm	4 s	0.68 wt%
B 6	ZnO 70 nm	6 s	0.82 wt%
B 8	ZnO 70 nm	8 s	1.15 wt%
B 10	ZnO 70 nm	10 s	1.04 wt%
